# Synthesis and Application of an Aldazine-Based Fluorescence Chemosensor for the Sequential Detection of Cu^2+^ and Biological Thiols in Aqueous Solution and Living Cells

**DOI:** 10.3390/s16010079

**Published:** 2016-01-11

**Authors:** Hongmin Jia, Ming Yang, Qingtao Meng, Guangjie He, Yue Wang, Zhizhi Hu, Run Zhang, Zhiqiang Zhang

**Affiliations:** 1 Key Laboratory for Functional Material, Educational Department of Liaoning Province, University of Science and Technology Liaoning, Anshan 114051, China; jhongmin66@163.com (H.J.); yangming0721@163.com (M.Y.); Wangyue9088@163.com (Y.W.); huzhizhi@163.com (Z.H.); 2 Department of Forensic Medicine, Xinxiang Medical University, Xinxiang, He‘nan 453003, China; guangjiehe@163.com; 3 Department of Chemistry and Biomolecular Sciences, Macquarie University, Sydney, NSW 2109, Australia; run.zhang@mq.edu.au

**Keywords:** aldazine, chemosensing ensemble, biothiols, detection, fluorescence imaging

## Abstract

A fluorescence chemosensor, 2-hydroxy-1-naphthaldehyde azine (**HNA**) was designed and synthesized for sequential detection of Cu^2+^ and biothiols. It was found that **HNA** can specifically bind to Cu^2+^ with 1:1 stoichiometry, accompanied with a dramatic fluorescence quenching and a remarkable bathochromic-shift of the absorbance peak in HEPES buffer. The generated **HNA**-Cu^2+^ ensemble displayed a “turn-on” fluorescent response specific for biothiols (Hcy, Cys and GSH) based on the displacement approach, giving a remarkable recovery of fluorescence and UV-Vis spectra. The detection limits of **HNA**-Cu^2+^ to Hcy, Cys and GSH were estimated to be 1.5 μM, 1.0 μM and 0.8 μM, respectively, suggesting that **HNA**-Cu^2+^ is sensitive enough for the determination of thiols in biological systems. The biocompatibility of **HNA** towards A549 human lung carcinoma cell, was evaluated by an MTT assay. The capability of **HNA**-Cu^2+^ to detect biothiols in live A549 cells was then demonstrated by a microscopy fluorescence imaging assay.

## 1. Introduction

Chemosensors are molecules of abiotic origin that bind selectively and reversibly to the analyte of interest with concomitant change in one or more properties of the system [1,2,3], such as fluorescence [4], color [5] or redox potential [6]. Among of them, fluorescent chemosensors have several advantages over the other methods due to their sensitivity, specificity and real-time monitoring with fast response times [7,8,9]. Particularly, fluorescence chemosensors are convenient to image physiologically important ions and small-molecules by *in situ* methods. To date, an enormous amount of work has been done for the rational design of fluorescent chemosensor for ions and neutral analytes [10,11,12,13,14,15,16].

Biothiols, including cysteine (Cys), homocysteine (Hcy), and glutathione (GSH) play important roles in a myriad of vital cellular processes such as biological redox homeostasis, biocatalysis, metal binding and post translational modifications [17,18,19]. Specifically, it has been reported that Hcy is an essential biological molecule required for the growth of cells and tissues [20,21]. As the most abundant cellular thiol, GSH plays a central role in combating oxidative stress and maintaining redox homeostasis [22,23]. Cys is a semi-essential aminoacid, and its thiol side chain serves as a nucleophile in many enzymatic reactions [24,25]. Abnormal levels of cellular biothiols are implicated in a variety of diseases, such as leucocyte loss, psoriasis, liver damage, slowed growth, asthma, cancer and AIDS [26,27]. At elevated levels in plasma, Hcy is a well-known risk factor for Alzheimer’s disease, folate and cobalamin (vitamin B12) deficiencies, and cardiovascular diseases [28]. Variations in GSH levels are associated with chronic diseases such as cancer, neurodegenerative diseases, cystic fibrosis (CF), HIV, and aging [29,30]. Cys deficiency is involved in many syndromes, for instance, slow growth in children, liver damage, skin lesions and weakness [31]. Elevated levels of Cys are associated with neurotoxicity, which has been demonstrated in animals with immature blood-brain barriers and in cultured neurons *in vitro* [32].

In view of their importance, safe, highly selective and sensitive detection methods for biothiols in living systems have very desirable [33]. In the past decades, continuous efforts have been made with regard to chemical and physical methods for the detection of biothiols, including HPLC, capillary electrophoresis, and optical assay mass spectrometry, electrochemical assay, and surface-enhanced Raman scattering (SERS) [34,35,36,37]. However, these methods generally have some limitations, e.g., high equipment costs, complexity, and time consuming sample processing or assays, which make them impractical for applications such as high-throughput clinical tests or research purposes [38]. Fluorescence-based methods using responsive chemosensors have long been recognized as one of the most promising techniques for thiol detection due to their high sensitivity, selectivity, simplicity of operation and potential application in living cell imaging. In the past few years, various fluorescent chemosensors to detect biothiols have been developed by exploiting diverse reaction mechanisms, including Michael addition [39,40], cyclization reactions with aldehyde [41,42], cleavage reactions by thiols [43,44], and others [45,46].

Recently, more attention has been paid to indicator displacement assays based on a simple competition mechanism between an indicator and analytes [47,48]. For the displacement strategy, the receptor is non-covalently attached to the indicator (cation) forming a so-called chemosensing ensemble, which is non-fluorescent due to metal ion-induced fluorescence quenching. Further addition of small-molecules or anions, however, may remove the metal ion and release the fluorophore-ligand into solution, with the revival of fluorescence [49]. It is well known that Cu^2+^ is a fluorescence quencher due to its notorious paramagnetic nature [50]. Moreover, Cu is well known as a “soft” metal with high affinity to –SH groups in biothiol side chains. Accordingly, based on exploitation of Cu^2+^ as an “ON–OFF–ON’’ signaling motif, sensing ensemble systems comprising multifunctional fluorophores ligated to the Cu^2+^ centre have been developed for the selective detection of biothiols [51].

**Scheme 1:**
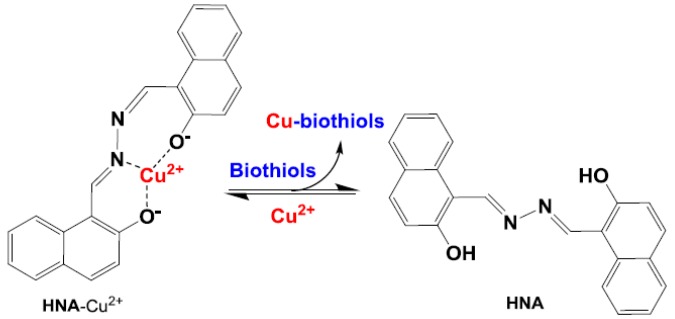
Schematic illustration of the design and sensing mechanism of **HNA**-Cu^2+^ ensemble for the reversible detection of bioactive thiols.

In this context, we report an aldazine-based fluorescence ligand, 2-hydroxy-1-naphthaldehyde azine (**HNA**) prepared by a straightforward condensation reaction. The emission signal of **HNA** was selectively quenched by Cu^2+^ via forming a **HNA-**Cu^2+^ complex, and was exclusively recovered followed by addition of thiols (Scheme 1). This reversible “ON–OFF–ON” response provides a convenient and practical way for the detection of biothiols in aqueous media and biological samples. The photophysical properties and biothiols recognition behaviors of **HNA-**Cu^2+^ have been investigated in detail through UV-Vis absorption spectra, fluorescence spectra and microscopy fluorescence images in biological cells.

## 2. Experimental Section

### 2.1. Chemicals

2-Hydroxy-1-naphthaldehyde, hydrazine hydrate and 4-diethylaminosalicylaldehyde were purchased from Sinopharm Chemical Reagent Co., Ltd. (Shanghai, China). Reagents and solvents were of A. R. grade and used without further purification unless otherwise noted. Fresh stock solution of metal ions (nitrate salts, 20 mM) and thiols, amino acids (20 mM) in H_2_O were prepared for further experiments.

### 2.2. Apparatus

^1^H-NMR and ^13^C-NMR spectra were recorded with an AVANCE 500 MHz spectrometer (Bruker, Fällanden, Switzerland) with chemical shifts reported as ppm (in DMSO, TMS as internal standard). API mass spectra were recorded on a 1100LC/MSD spectrometer (HP, Palo Alto, CA, USA). The elemental analyses of C, H, N and O were performed on a EL III elemental analyzer (Vario, Frankfurt, Germany). The melting point of **HNA** was measured by DSC4000 differential scanning calorimetry (Perkin Elmer, Waltham, MA, USA). Fluorescence spectra were determined with a LS 55 luminescence spectrometer (Perkin Elmer). The absorption spectra were measured with a Lambda 900 UV/VIS/NIR spectrophotometer (Perkin Elmer). Fluorescent live cell images were acquired on a Ti-S inverted fluorescence microscope with an objective lens (×20) (Nikon, Tokyo, Japan). Excitation with blue light was used for fluorescence imaging.

### 2.3. General Procedures of Spectra Detection

Stock solutions of **HNA** was prepared in DMF-HEPES buffer (20 mM, pH = 7.4, 3:7 v/v). The excitation wavelength for **HNA** was 411 nm. Before spectroscopic measurements, the solution was freshly prepared by diluting the high concentration stock solution to corresponding solution (10 μM). Each time a 3 mL solution of chenosensor was filled in a quartz cell of 3 cm optical path length, and different stock solutions of cations were added into the quartz cell gradually by using a micro-syringe. The volume of cationic stock solution added was less than 100 μL with the purpose of keeping the total volume of testing solution without obvious change. **HNA**-Cu^2+^ solution for thiols and amino acids detection was prepared by addition of 3.0 equiv. of Cu^2+^ to **HNA** (10 μM) solution in DMF-HEPES buffer (20 mM, pH = 7.4, 3:7 v/v). The quantum yields were determined according to a reported procedure using fluorescein as standard (Φ_f_ = 0.85 in 0.1 N NaOH aqueous solutions) [52].

### 2.4. Association Constant Calculation

Generally, for the formation of 1:1 complexation species formed by the chemosensor compound and the guest cations, the Benesi-Hildebrand equation used is as follows [53]:

$$\frac{1}{F_{0}-F}=\frac{1}{K_{a}(F_{0}-F_{\min})[{\mathrm{Cu}}^{2+}]}+\frac{1}{F_{0}-F_{\min}}$$

where *F* and *F*_0_ represent the fluorescence emission of **HNA** in the presence and absence of Cu^2+^, respectively, *F*_min_ is the saturated emission of **HNA** in the presence of excess amount of Cu^2+^; [Cu^2+^] is the concentration of Cu^2+^ ion added, and *K*_a_ is the binding constant.

### 2.5. Synthesis and Characterization the Fluorescent Chemosensor **HNA**

The synthesis of **HNA** was carried out according to the previously reported method [54]. To a solution of 2-hydroxy-1-naphthaldehyde (0.172 g, 0.5 mmol) in methanol (10 mL), hydrazine (0.5 equiv.) in methanol (10 mL) was added slowly at room temperature. The stirred reaction mixture was heated to reflux for 6 h. The formed yellow precipitate was filtered, washed with methanol and then dried under vacuum to obtain **HNA** in 87% yield. Yellow solid, m.p. 310 °C, ^1^H-NMR (DMSO-d_6_, 500 MHz) δ(ppm): 12.89 (s, 1H), 10.00 (s, 1H), 8.667 (d, *J* = 10.5 Hz, 1H), 8.046 (d, *J* = 11.5 Hz, 1H), 7.929 (d, *J* = 10 Hz, 1H), 7.628 (t, *J* = 9.5 Hz, 1H), 7.452 (t, *J* = 9.25 Hz, 1H), 7.294 (d, *J* = 11 Hz, 1H). ^13^C-NMR (DMSO-d_6_, 125 MHz) δ (ppm): 163.12, 151.29, 148.16, 140.61, 137.33, 136.26, 124,54, 122.12, 114.44, 110.64, 107.88. ESI-mass spectra (positive mode, *m*/*z*): Calcd for C_22_H_17_N_2_O_2_: 341.1290 [**HNA** + H]^+^; Found: 341.1290. Elem Anal: Calcd for **HNA**: C, 77.63; H, 4.74; N, 8.23; O, 9.40. Found: C 77.68; H 4.75; N 8.21; O 9.42.

### 2.6. Fluorescence Imaging of Biothiols in A549 Cells

The human lung cancer cell A549 line was acquired from the Cell Bank of the Chinese Academy of Sciences (Shanghai, China) and cultured in F12K medium, supplemented with 10% (v/v) fetal bovine serum (FBS), penicillin (100 μg/mL), streptomycin sulphate (100 μg/mL). Cells were cultured in a humidified 37 °C, 5% CO_2_/95% air (v/v) incubator. The growth medium was changed every two days. The cells were grown to 80% confluence prior to experiment. For the fluorescence microscope imaging, cells were typically seeded at a density of 5 × 10^4^ cells/mL in a 22 mm cover-glass bottom culture dishes (ProSciTech, Shanghai, China). A549 cells were incubated with **HNA**-Cu^2+^ (10 μM in PBS medium) for 30 min at 37 °C. After removal of the growth medium, the cells were imaged by inverted fluorescence microscopy. As a control experiment, the A549 cells were pre-treated with an excess of N-ethylmaleimide (NEM, 1 mM) for 30 min and then treated with 10 μM of **HNA**-Cu^2+^ for another 30 min, and subjected to fluorescence microscope imaging.

## 3. Results and Discussion

### 3.1. Spectroscopic Studies of **HNA** in the Presence of Cu^2+^

**HNA** was easily synthesized by a straightforward synthetic route. Briefly, 2-hydroxy-1-naphthaldehyde was treated with 0.5 equv. of hydrazine in methanol solution at room temperature to form the Schiff base ligand **HNA** in quantitative yield. The structure of **HNA** was confirmed by NMR, mass spectra and elemental analysis. Early studies revealed that salicylaldehyde-based hydrazone framework is a typical scaffold for the construction of Cu^2+^ fluorescent chemosensor [55]. In the present work, **HNA** displayed excellent selectivity for Cu^2+^ in the presence of other competing cations with observable changes in UV-Vis and fluorescence spectra in HEPES buffer. By virtue of the extremely high copper-sulfur affinity, the *in situ* generated **HNA**-Cu^2+^ fluorescence ensemble was expected to act as a potential chemosensor for biothiol determination *via* a Cu^2+^ displacement approach.

The stability of the unique ligand, **HNA** was firstly confirmed by the measurement of fluorescence intensities in DMF-HEPES buffer (20 mM, pH = 7.4, 3:7 v/v). As shown in Figure S1, no obvious fluorescent intensities changes were observed even after 30 h, indicating that **HNA** is stable under the test conditions. For the biological application of **HNA**, the sensing should be operated in a physiological range of pH. Therefore, the effect of pH on fluorescence intensities of **HNA** and **HNA**-Cu^2+^ was evaluated in water with different pH levels. As shown in Figure S2, the suitable pH range for Cu^2+^ determination was confirmed to be pH 6.0–10.0, suggesting that the proposed chemosensor is suitable for application under physiological conditions.

**Figure 1:**
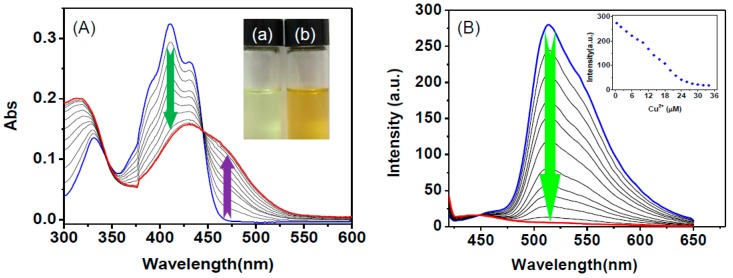
(**A**) UV-Vis absorption spectra of **HNA** (10 μM) in the presence of increasing amount of Cu^2+^ (0–35 μM) in DMF-HEPES buffer (20 mM, pH = 7.4, 3:7 v/v). Insert: Colorimetric changes of **HNA** without (**a**) and with (**b**) the addition of Cu^2+^ in HEPES buffer; (**B**) Fluorescence spectra of **HNA** (10 μM) in DMF-HEPES buffer (20 mM, pH = 7.4, 3:7 v/v) in the presence of different amounts of Cu^2+^ (0–35 μM). Insert: normalized fluorescence intensities of **HNA** (10 μM) at 513 nm as a function of Cu^2+^ (0–35 μM). Excitation was performed at 411 nm.

The specific complexation of **HNA** with Cu^2+^ was first investigated by the UV-Vis absorption spectrum in HEPES buffer. As shown in Figure 1A, **HNA** displays an intense absorption band centered at 411 nm. Upon addition of increasing amounts of Cu^2+^ (0–3.5 equiv) to **HNA** in HEPES buffer, the maximum absorption band of **HNA** centered at 411 nm was gradually reduced, and a new absorption band appeared at 457 nm (Figure 1A). The changes of absorbance reached to the maximum value upon addition of 3.0 equiv. of Cu^2+^. In addition, an obvious color changes from yellowish to yellow was observed for **HNA** solution when the addition of Cu^2+^ (Figure 1A inset), suggesting that the **HNA** could be used as a chemosensor for the detection of Cu^2+^ by the naked eye.

The fluorescence titration of **HNA** towards representative metal ions and its selectivity for Cu^2+^ in DMF-HEPES buffer (20 mM, pH = 7.4, 3:7 v/v) were further investigated. As shown in Figure 1B. **HNA** displayed a strong green fluorescence emission at 513 nm (Φ_1_ = 0.535). The fluorescence emission at 513 nm of **HNA** (10 μM) could be more than 95% quenched in the presence of 35 μM Cu^2+^ (Φ_2_ = 0.092), which could be ascribed to the aparamagnetic quenching effect of Cu^2+^ [56]. Job’s plot reveals that **HNA** forms a 1:1 stoichiometry complex with Cu^2+^ (Figure S3). According to linear Benesie-Hildebrand expression, the measured fluorescence intensity [1/(F_0_–F)] at 513 nm varied as a function of 1/[Cu^2+^] in a linear relationship (R^2^ = 0.9988), which further indicating the 1:1 stoichiometry between Cu^2+^ and **HNA** (Figure S4) [57]. The association constant of **HNA** with Cu^2+^ in HEPES buffered was calculated to be 1.59 × 10^5^ M^−1^. The fluorescence intensity changes of **HNA** at 513 nm exhibited a linear correlation to Cu^2+^ in the concentration range from 0 to 3.5 μM (R^2^ = 0.9957) (Figure S5). The detection limit for Cu^2+^ was estimated to be 15 nM based on a 3*σ*/*slope* under the experimental conditions used here [58], which is below the maximum permissive level of Cu^2+^ in drinking water (20 μM) set by the U.S. Environmental Protection Agency [59].

**Figure 2:**
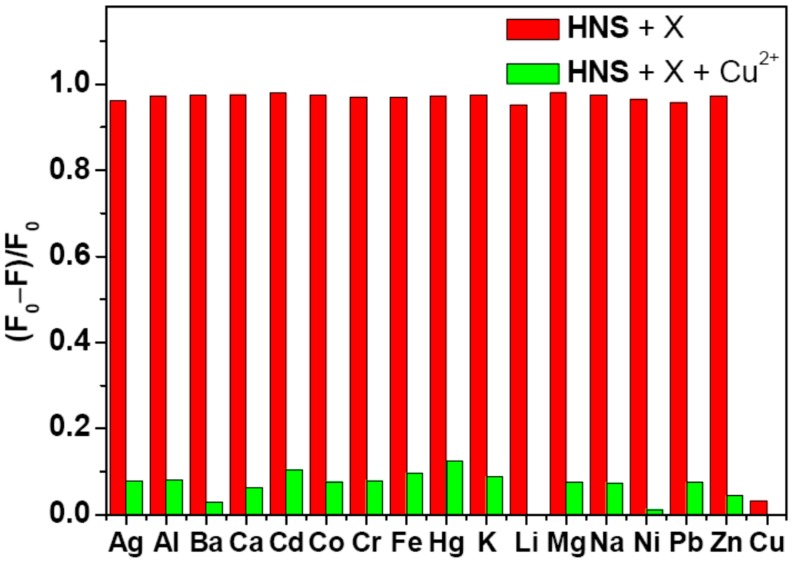
Normalized fluorescence responses of **HNA** (10 μM) to various cations in DMF-HEPES buffer (20 mM, pH = 7.4, 3:7 v/v). The red bars represent the emission changes of **HNA** in the presence of cations of interest (all are 30 μM). The green bars represent the changes of the emission that occurs upon the subsequent addition of 30 μM of Cu^2+^ to the above solution. The intensities were recorded at 513 nm, excitation at 411 nm.

The fluorescence response of **HNA** towards various metals ions was then examined in HEPES buffer. As shown in Figures S6 and S7, no obvious changes in UV-Vis absorption and color were observed when the **HNA** in HEPES solution was added other competitive metal ions, including Fe^3+^, Hg^2+^, Cd^2+^, Pb^2+^, Zn^2+^, Ni^2+^, Co^2+^, Mn^2+^, Cr^3+^, Ag^+^, Ca^2+^, Mg^2+^, Ba^2+^, Li^+^, K^+^, and Na^+^, indicating that **HNA** could be used as a potential candidate for Cu^2+^-specific colorimetric chemosensor in aqueous media. Furthermore, **HNA** also showed a selective fluorescence quenching only with Cu^2+^ among the various metal ions under similar testing conditions. Upon addition of competitive cations (30 μM) to the solution of **HNA** (10 μM), the quenching rate of the emission intensities at 513 nm was evaluated, and the result was displayed in Figure 2. It was found that no significant changes of fluorescence intensities occurred in the presence of Fe^3+^, Hg^2+^, Cd^2+^, Pb^2+^, Zn^2+^, Ni^2+^, Co^2+^, Mn^2+^, Cr^3+^, Ag^+^ and Ca^2+^, Mg^2+^, Ba^2+^, Li^+^, K^+^, Na^+^. In addition, it is notable that the fluorescence response of **HNA** to a mixture containing all of the chosen metal ions is similar to that to Cu^2+^ only. The result indicates that Cu^2+^-specfic responses were not disturbed by competitive metal ions.

### 3.2. Spectra Recognition of Thiols by **HNA**-Cu^2+^ Ensemble

**Figure 3:**
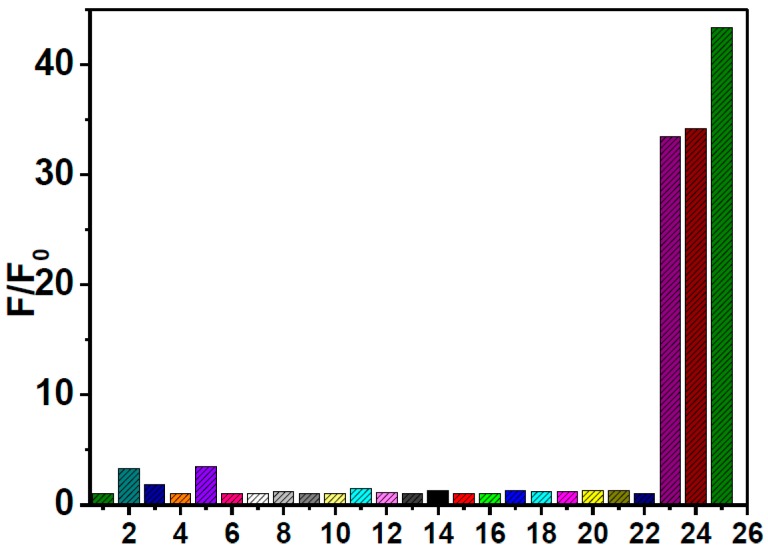
Normalized fluorescence responses of **HNA**-Cu^2+^ (10 μM) to various amino acid (40 μM) in DMF-HEPES buffer (20 mM, pH = 7.4, 3:7 v/v). 1. II-Leu, 2.Ala, 3. Arg, 4. Asn, 5. Asp, 6. Gln, 7. Glu, 8. Gly, 9. His,10. Leu, 11. Lys, 12. Met, 13. Phe, 14. Pro, 15. Ser, 16. Thr, 17. Try, 18. Val, 19. Methyl mercaptan, 20. Ethyl mercaptan, 21. 1,3-Dimercaptopropane, 22. S^2−^, 23. GSH, 24. Cys, 25. Hcys. The intensities were recorded at 513 nm, excitation at 411 nm.

Considering the high copper-sulfur affinity, we are encouraged to envision that the obtained sluggish **HNA**-Cu^2+^ system fluorescence could be employed as a promising chemosensor for fluorescence “OFF–ON” detection of biothiols *via* Cu^2+^ displacement approach. To confirm our assumption, the selectivity of the **HNA**-Cu^2+^ ensemble for a variety of relevant biological species such as natural aminoacids and bioactive thiols was firstly evaluated by fluorescence titration. The **HNA**-Cu^2+^ solution was prepared *in situ* by addition of 3.0 equiv. of Cu^2+^ to **HNA** (10 μM) in DMF-HEPES buffer (20 mM, pH = 7.4, 3:7 v/v). Figure 3 shows the ratio of fluorescence intensity enhancement (F/F_0_) at 513 nm upon addition of various bioactive species (40 μM). A 47.8-fold fluorescence enhancement of **HNA**-Cu^2+^ was observed when 40 μM Hcy was finally added (Φ_3_ = 0.486). Furthermore, the addition of Cys and GSH into **HNA**-Cu^2+^ in HEPES solution led to significant enhancement in fluorescence intensity of **HNA**-Cu^2+^: *i.e.*, Cys (42.2-fold) and GSH (36.2-fold). Concomitantly, the quantum yield of **HNA**-Cu^2+^ increased to 0.464 (Φ_4_) and 0.422 (Φ_5_), respectively. In contrast, other aminoacids such as II-Leu, Ala, Arg, Asn, Asp, Gln, Glu, Gly, His, Leu, Lys, Met, Phe, Pro, Ser, Thr, Try and Val induced negligible fluorescence intensity changes. In addition, no significant changes of the fluorescence intensities of **HNA**-Cu^2+^ were observed in the presence of other low-molecular weight thiols and sulfide, such as methyl mercaptan, ethyl mercaptan, 1,3-dimercaptopropane and S^2−^. These results demonstrate that **HNA**-Cu^2+^ has an excellent selectivity toward biological thiols, including Hcy, Cys, GSH. The co-operative effect of the thiol–amino–carboxylic acid moiety as a ligand to Cu^2+^ could be a possible factor for the selectivity to biological thiols of **HNA**-Cu^2+^.

**Figure 4:**
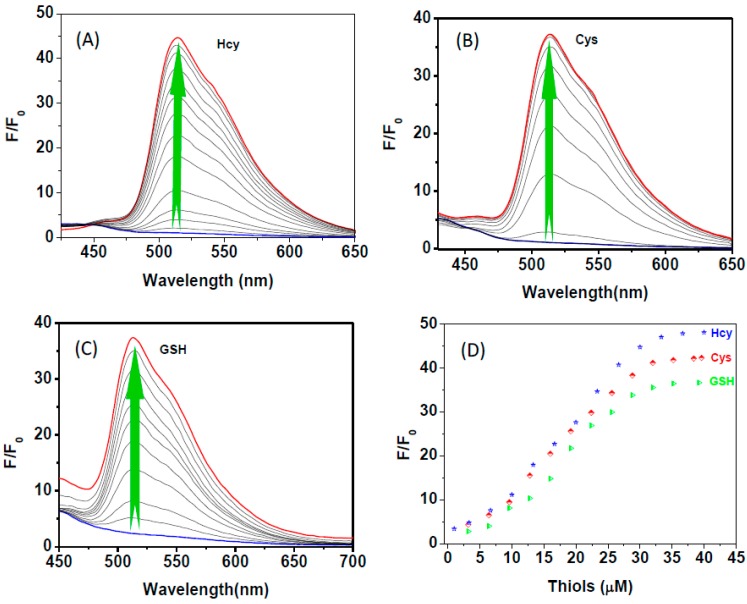
Fluorescence emission spectra of **HNA**-Cu^2+^ (10 μM) in DMF-HEPES buffer (20 mM, pH = 7.4, 3:7 v/v) in the presence of 0–40 μM (A) Hcy; (**B**) Cys and (**C**) GSH. (**D**) The plots of the fluorescence intensities of **HNA**-Cu^2+^ (10 μM) observed at 513 nm *versus* thiol concentration (0–40 μM). Excitation was performed at 411 nm.

**Figure 5:**
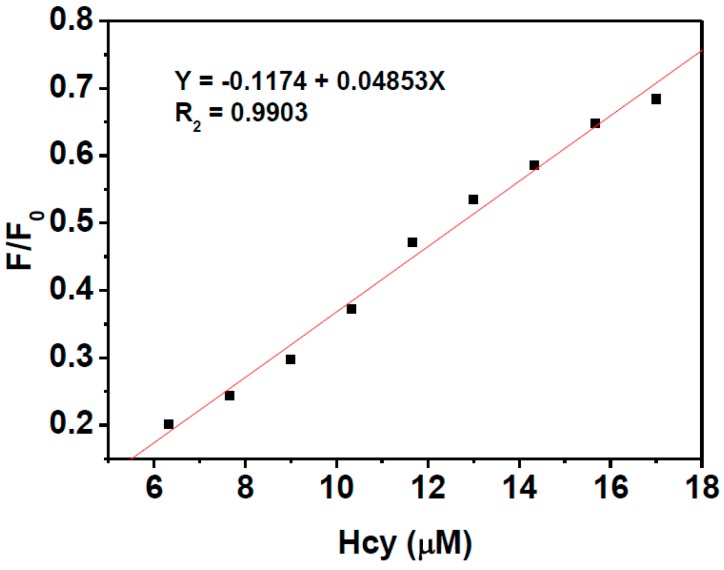
The linear responses of **HNA**-Cu^2+^ (3 μM) *versus* low concentration Hcy (0–17 μM) at 513 nm. Excitation was performed at 411 nm.

The turn on fluorescence response of **HNA**-Cu^2+^ towards biothiols was then investigated by addition of Cys, Hcy, and GSH to HNA-Cu^2+^ in HEPES solution. As shown in Figure 4A,D, upon addition of increasing amounts of Hcy, the fluorescence intensity of **HNA**-Cu^2+^ solution increased gradually and the emission intensity at 513 nm reached a maximum level when 40 equiv. of Hcy was added. Similar fluorescence recovery results were observed in the tests of Cys and GSH (Figure 4B–D). The relative fluorescence intensity of **HNA**-Cu^2+^ is linearly proportional to Hcy concentration of 0–3.5 μM, and the detection limit for Hcy was estimated to be as low as 1.5 μM (Figure 5). The detection limits of **HNA**-Cu^2+^ for Cys and GSH were calculated to be 1.0 μM and 0.8 μM, respectively (Figures S8 and S9). These results demonstrate that **HNA**-Cu^2+^ is sensitive enough for its practical applications in determination of biothiols in biological fluids [60,61,62].

**Figure 6:**
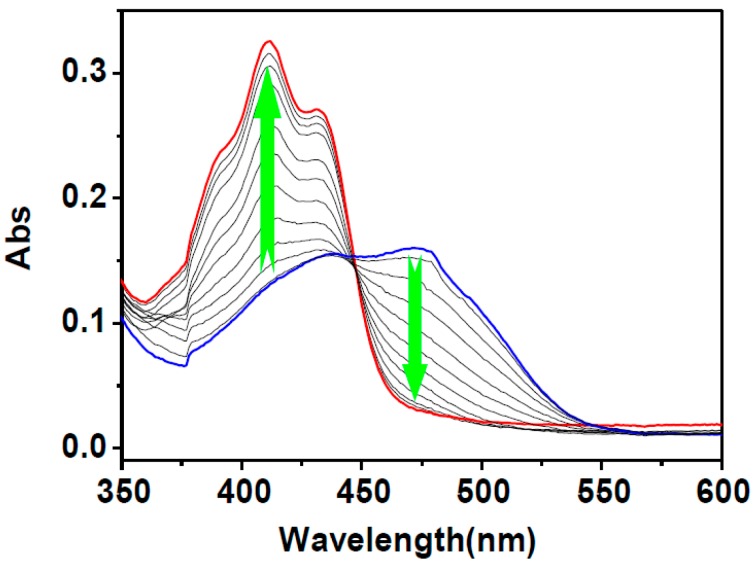
UV-Vis absorption spectra of **HNA**-Cu^2+^ (10 μM) in the presence of increasing amount of Hcy (40 μM) in DMF-HEPES buffer (20 mM, pH = 7.4, 3:7 v/v).

**Figure 7:**
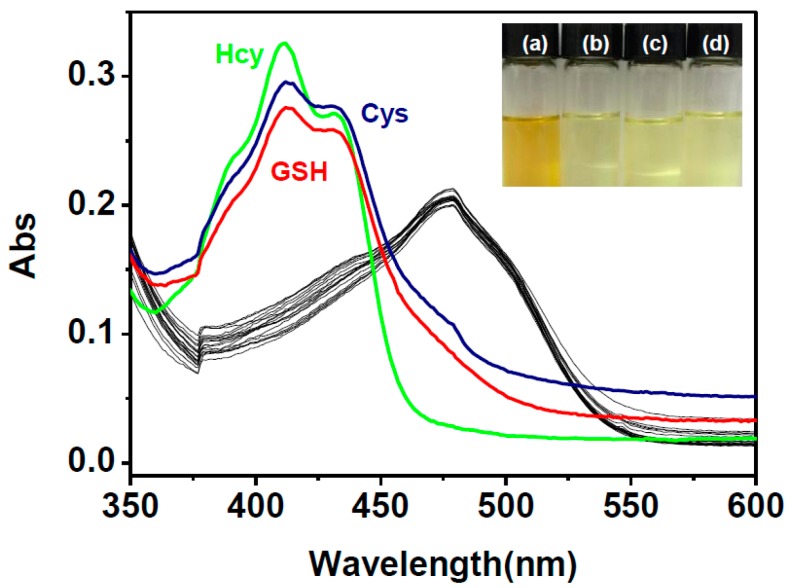
Absorption spectra of **HNA**-Cu^2+^ (10 μM) in DMF-HEPES buffer (20 mM, pH = 7.4, 3:7 v/v) upon addition of various amino acid and thiols (40 μM). Insert: Colorimetric changes of **HNA**-Cu^2+^ (**a**) in the presence of (**b**) Hcy, (**c**) Cys snd (**d**) GSH in HEPES buffer.

The capability of **HNA**-Cu^2+^ to detect biothiols in HEPES aqueous buffer was also evaluated by the measurement of the corresponding UV-Vis absorption spectra. As shown in Figure 6, upon addition of increasing amounts of Hcy, the absorption band at 475 nm faded gradually with the concomitant increase of the absorption at 411 nm. The final absorption spectrum is identicaaal to that of **HNA** under identical conditions. Upon the addition of Cys and GSH similar UV-Vis spectroscopic changes of **HNA**-Cu^2+^ were observed to those encountered using Hcy (Figures S10 and S11). Meanwhile, the yellow solution of **HNA**-Cu^2+^ returned to yellowish upon the addition of biothiols, indicating that **HNA**-Cu^2+^ ensemble can serve as a “naked-eye” thiol indicator in aqueous media (Figure 7). In addition, the selectivity of **HNA**-Cu^2+^ ensemble towards biothiols was further investigated by UV-Vis spectroscopy. As shown in Figure 7, changes of UV-Vis spectra only occurred upon the addition of biothiols, including Hcy, Cys, and GSH, demonstrating that **HNA**-Cu^2+^ has an excellent selectivity toward thiols.

**Figure 8:**
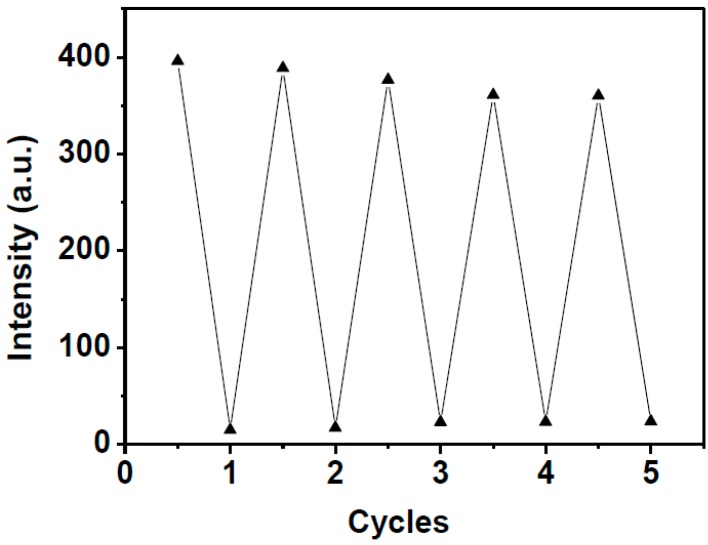
Fluorescent intensities of **HNA**-Cu^2+^ (10 μM) at 513 nm in DMF-HEPES buffer (20 mM, pH = 7.4, 3:7 v/v) upon the alternate addition of Hcy/Cu^2+^ with several concentrations ratio (0:0, 20:0, 20:40, 80:40, 80:160, 160:160, 160:320, 320:320, 320:640, 640:640 μM, respectively). Excitation at 411 nm.

Reversibility is also one of the most important factors that should be considered for the development of chemosensors for the detection of analytes in practical applications. In the present work, the reversibility of the **HNA**-Cu^2+^ ensemble in sensing biothiols has been demonstrated by carrying out alternate cycles of titration of **HNA** with Cu^2+^ followed by biothiols. As shown in Figure 8, by the sequential addition of Cu^2+^/Hcy, it was found that “ON−OFF−ON” changes in the fluorescence intensity of **HNA**-Cu^2+^ at 513 nm can be repeated more than five times, indicating that **HNA**-Cu^2+^ can be developed as a reversible fluorescence chemosensor for Hcy detection. As expected, the fluorescence emission also could be turned off and on repeatedly with the alternate addition of Cu^2+^ and Cys or GSH, indicating that the **HNA**-Cu^2+^ system can be developed as a regeneratable fluorescence turn-on probe for biothiols (Figures S12 and S13).

ESI-MS analysis of **HNA** sequentially in the presence of Cu^2+^ and thiols was further undertaken to verify the fluorescence “ON−OFF−ON” reversible interconversion states. ESI-MS of **HNA** displayed a molecular-ion peak [**HNA** − H]^+^ at *m*/*z* 339.1 (Figure S14). When three equiv. of Cu^2+^ were added into the **HNA** solution, the peak at *m*/*z* 401.08 was appeared, which can be assignable to a [**HNA** + Cu^2+^]^+^ species (Figure S15). This result confirmed formation of a 1:1 stoichiometry complex between **HNA** and Cu^2+^. With further addition of thiols, e.g., 40 μM Hcy, the peaks at *m*/*z* 401.08 of the **HNA**-Cu^2+^ ensemble disappeared, and the peak at *m*/*z* 339.05 related to the native **HNA** was observed again (Figure S16), indicating that the binding between Hcy and Cu^2+^ led to the release of **HNA**.

### 3.3. Living-Cell Fluorescence Imaging Studies

**Figure 9:**
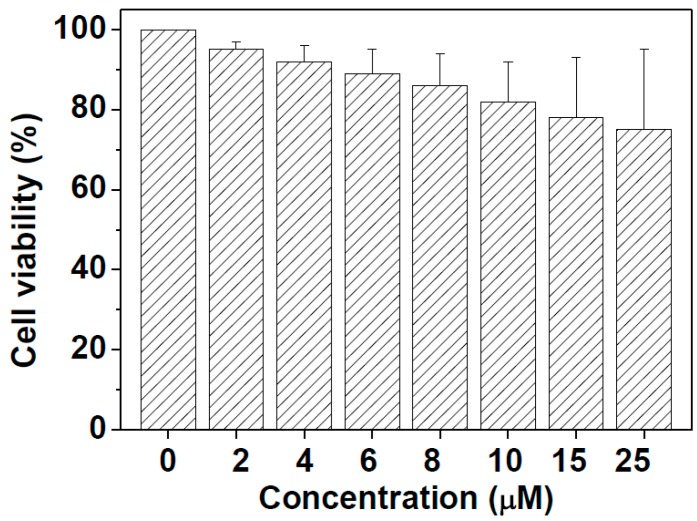
Cell viability values (%) estimated by a MTT proliferation test *versus* incubation concentrations. A549 cells were incubated in the **HNA**-containing culture medium at 37 °C in a 5% CO_2_ incubator for 24 h.

Prior to the microscopy imaging of biothiols in live cells, the long-term cytotoxicity of **HNA** to the human lung cancer cell A549 was evaluated using the MTT assay method [63,64]. Figure 9 presents the results of A549 cells incubated with different concentrations of **HNA** (0, 2, 4, 6, 8, 10, 15 and 25 μM) for 24 h. A549 cell viabilities remained approximately at 78% even at the high concentration of 25 μM for 24 h. The results demonstrated that **HNA** has low cytotoxicity both at low and high concentration.

**Figure 10:**
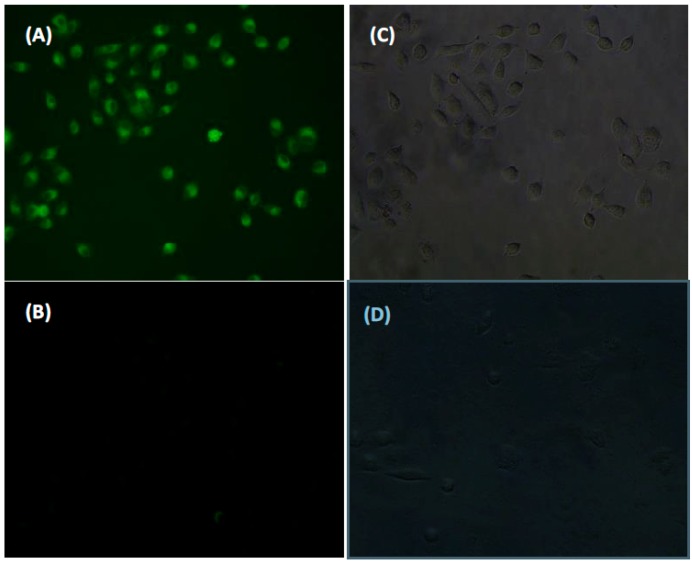
Live cell imaging of human lung cancer cell A549 at 37 °C. (**A**) A549 cells incubated with 10 μM **HNA**-Cu^2+^ for 30 min; (**B**) A549 cells were pre-incubated with 1 mM NEM for 30 min and then treated with 10 μM **HNA**-Cu^2+^ for another 30 min; (**C**) and (**D**) are the corresponding brightfield images. Excitation with blue light.

Typically, free intracellular Cys concentrations are on the order of 30–200 μM, and GSH is the most abundant among the small intracellular molecular thiols (1–10 mM). To demonstrate the capability of **HNA**-Cu^2+^ ensemble for the detection of biothiols in living cells, fluorescence microscopy imaging studies were conducted in the human lung cancer cell A549. The A549 cells were incubated with a 10 μM solution of **HNA**-Cu^2+^ in PBS for 30 min in a 37 °C incubator. Then the cells were washed with PBS three times and mounted on a microscope stage. As shown in Figure 10, the cells displayed strong green fluorescence emission. However, in a control experiment, cells were pre-treated with *N*-ethylmaleimide (NEM, 1 mM, a trapping reagent for thiol species), followed by the incubation of cells with **HNA**-Cu^2+^. A sluggish fluorescence pattern was exhibited inside the cells, indicating the specificity of **HNA**-Cu^2+^ for biological thiols over other bioactive analytes in living cells.

## 4. Conclusions

In conclusion, we have developed an aldazine-based fluorescence chemosensor, **HNA**, for the sequential detection of Cu^2+^ and biological thiols (Hcy, Cys and GSH) in aqueous solution and living cells. The binding of Cu^2+^ and **HNA** was found to follow a 1:1 stoichiometry, accompanied with a significant fluorescence quenching. The quenched fluorescence of the HNA-Cu^2+^ ensemble could be recovered upon the addition of thiols, realizing the detection of thiols by utilizing Cu^2+^ displacement approach. Fluorescence microscopy imaging suggested that HNA-Cu^2+^ ensemble has potential as a powerful tool for the detection of thiols in living cells. In summary, the success of this chemosensing ensemble not only provides a robust approach to detect biothiols in live cells, but also extends the development of displacement strategy-based fluorescence chemosensors.

## Supplementary Files

Supplementary File 1
