# Supplementary material for: Synthesis and Application of an Aldazine-Based Fluorescence Chemosensor for the Sequential Detection of Cu2+ and Biological Thiols in Aqueous Solution and Living Cells
Source: Sensors (Basel). 2016 Jan 11;16(1):79. doi: 10.3390/s16010079 (PMC4732112; doi:10.3390/s16010079)
Supplement: Supplementary File 1 [file sensors-16-00079-s001.pdf]

Supplementary Materials: Synthesis and Application of an Aldazine-Based Fluorescence Chemosensor for the Sequential Detection of Cu^{2+} and Biological Thiols in Aqueous Solution and Living Cells

Hongmin Jia [1], Ming Yang [1], Qingtao Meng [1,*], Guangjie He [2], Yue Wang [1], Zhizhi Hu [1], Run Zhang [3] and Zhiqiang Zhang [1,*]

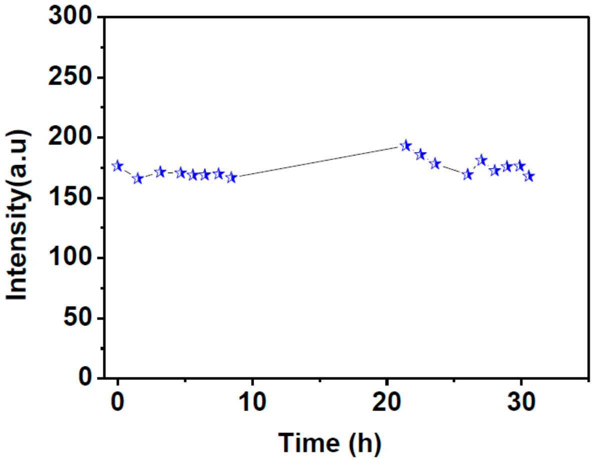

Figure S1. Fluorescence intensity of **HNA** (10 μM) at different time in DMF-HEPES buffer (20 mM, pH = 7.4, 3:7 v/v). The intensities were recorded at 513 nm, excitation at 411 nm.

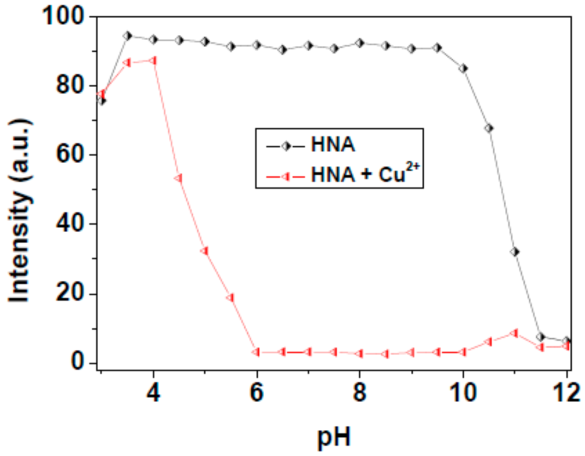

Figure S2. Variations of fluorescence intensity of **HNA** (10 μM) at 513 nm in aqueous solution with (bottom) and without (up) Cu^{2+} (0–20 μM) as a function of pH. Excitation at 411 nm.

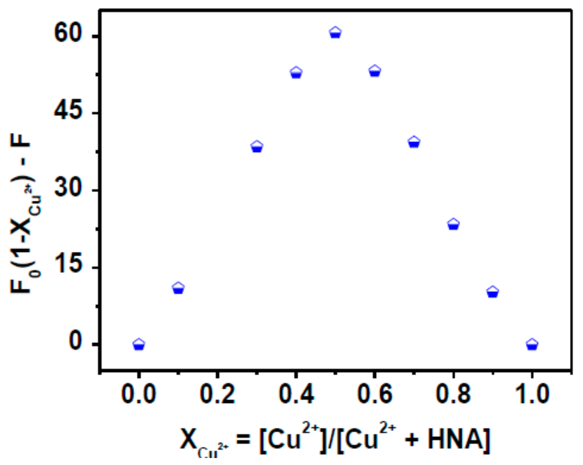

Figure S3. Job's plots according to the method for continuous variations. The total concentration of **HNA** and Cu^{2+} is 10 μM. The intensities were recorded at 513 nm, excitation at 411 nm.

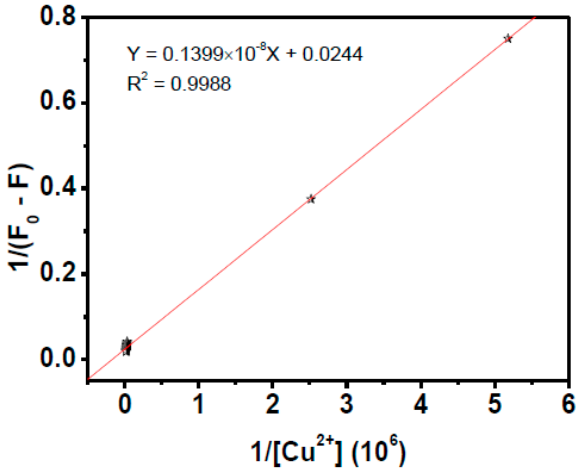

Figure S4. Benesi-Hildebrand plot of **HNA** (10 μM) based on 1:1 binding stoichiometry with Cu^{2+} ions. The intensities were recorded at 513 nm, excitation at 411 nm.

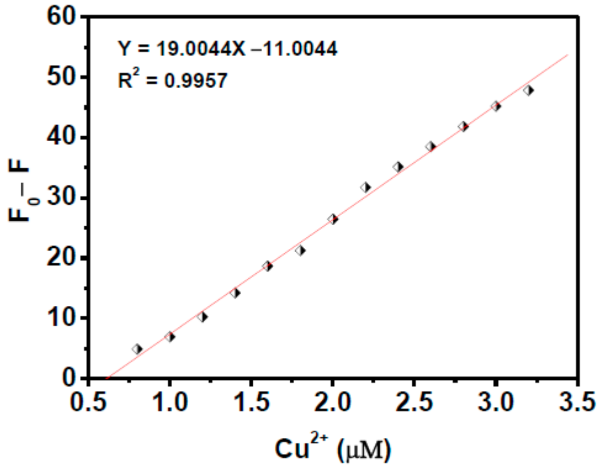

Figure S5. Linear relationship between fluorescence intensity of **HNA** (1 μM) at 513 nm *versus* the concentration of Cu^{2+} (0-3.5 μM) in DMF-HEPES buffer (20 mM, pH = 7.4, 3:7 v/v). Excitation was performed at 411 nm.

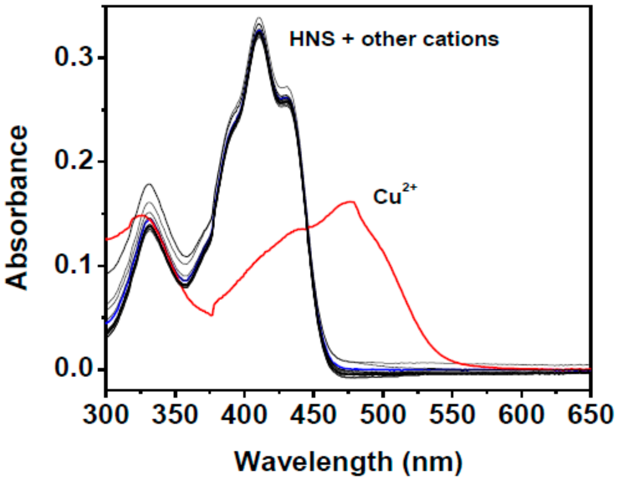

Figure S6. Absorption spectra of **HNA** (10 μM) in DMF-HEPES buffer (20 mM, pH = 7.4, 3:7 v/v) upon addition of various metal ions (30 μM).

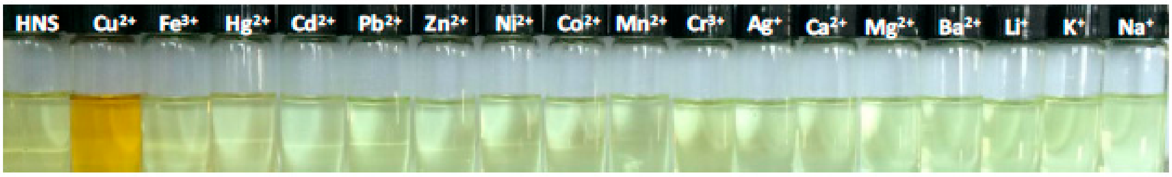

Figure S7. The colour changes of **HNA** (10 μM) in DMF-HEPES buffer (20 mM, pH = 7.4, 3:7 v/v) upon addition of various metal ions (30 μM).

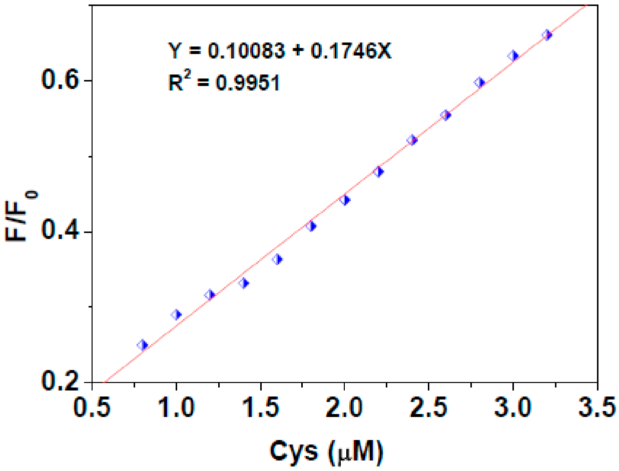

Figure S8. The linear fluorescence responses of **HNA**-Cu^{2+} (3 μM) *versus* low concentration Cys (0–3.3 μM) at 513 nm. Excitation was performed at 411 nm.

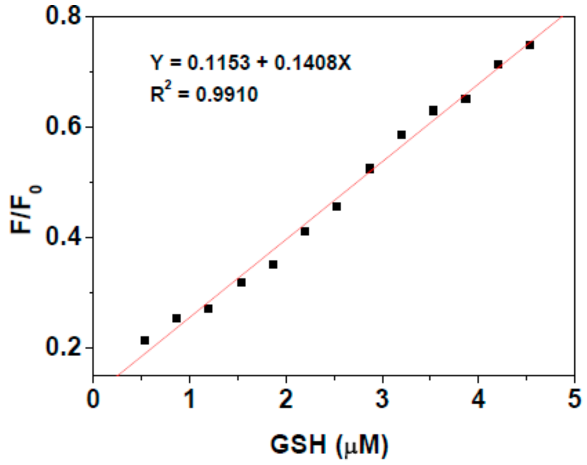

Figure S9. The linear fluorescence responses of **HNA**-Cu^{2+} (3 μM) *versus* low concentration GSH (0–4.6 μM) at 513 nm. Excitation was performed at 411 nm.

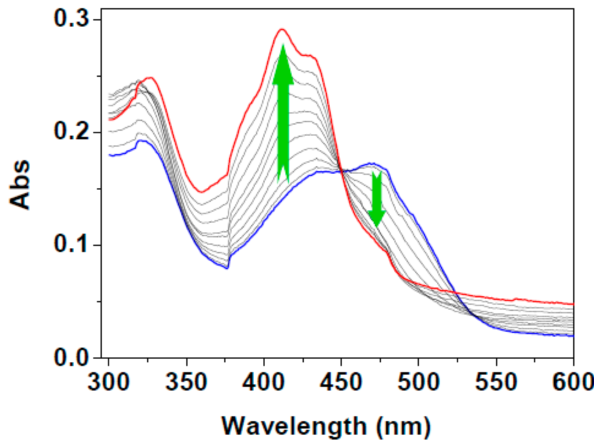

Figure S10. UV-Vis absorption spectra of **HNA**-Cu^{2+} (10 μM) in the presence of increasing amount of GSH (40 μM) in DMF-HEPES buffer (20 mM, pH = 7.4, 3:7 v/v).

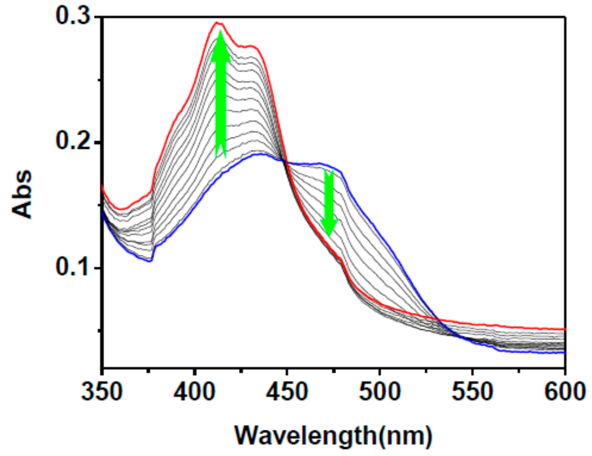

Figure S11. UV-Vis absorption spectra of **HNA**-Cu^{2+} (10 μM) in the presence of increasing amount of Cys (40 μM) in DMF-HEPES buffer (20 mM, pH = 7.4, 3:7 v/v).

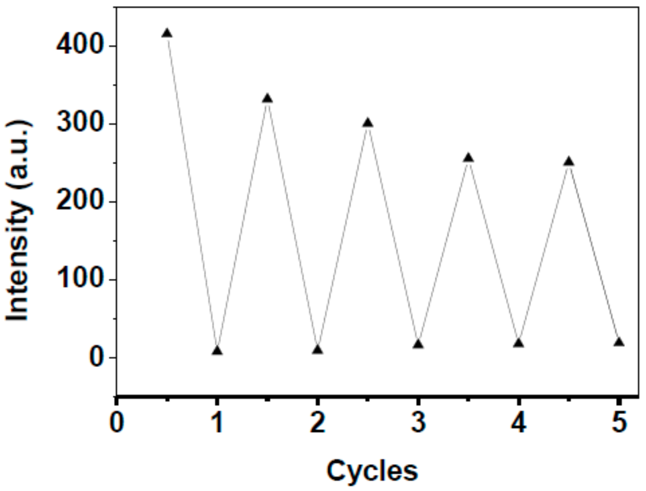

Figure S12. Fluorescence intensity of **HNA**-Cu^{2+} (10 μM) at 513 nm in DMF-HEPES buffer (20 mM, pH = 7.4, 3:7 v/v) upon the alternate addition of Cys/Cu^{2+} with several concentrations ratio (0:0, 20:0, 20:40, 80:40, 80:160, 160:160, 160:320, 320:320, 320:640, 640:640 μM, respectively). Excitation at 411 nm.

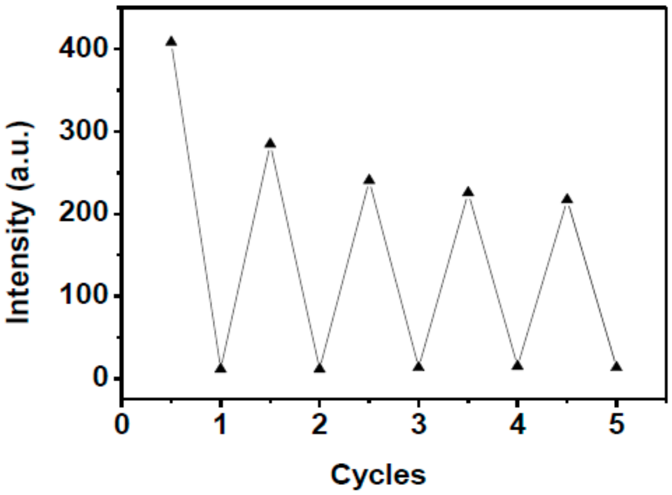

Figure S13. Fluorescence intensity of **HNA**-Cu^{2+} (10 μM) at 513 nm in DMF-HEPES buffer (20 mM, pH = 7.4, 3:7 v/v) upon the alternate addition of GSH/Cu^{2+} with several concentrations ratio (0:0, 20:0, 20:40, 80:40, 80:160, 160:160, 160:320, 320:320, 320:640, 640:640 μM, respectively). Excitation at 411 nm.

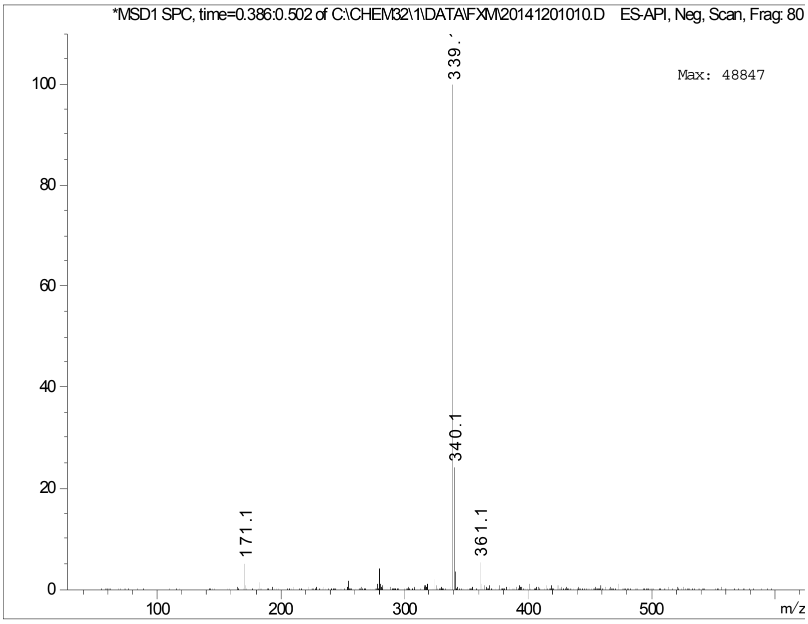

Figure S14. ESI-mass spectra of **HNA.**

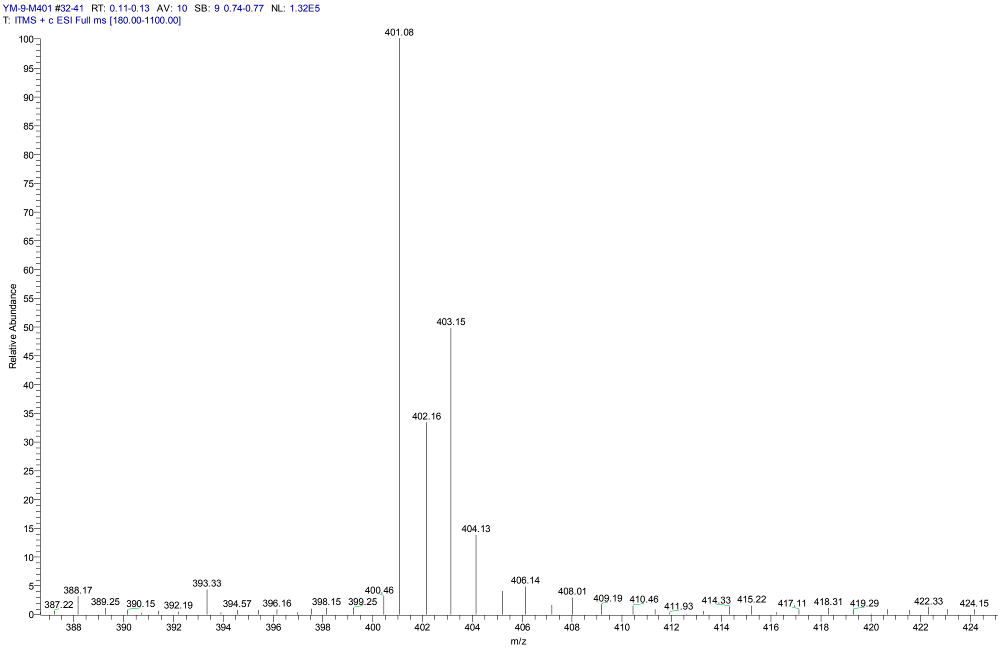

Figure S15. ESI-mass spectra of **HNA**-Cu^{2+} ensemble.

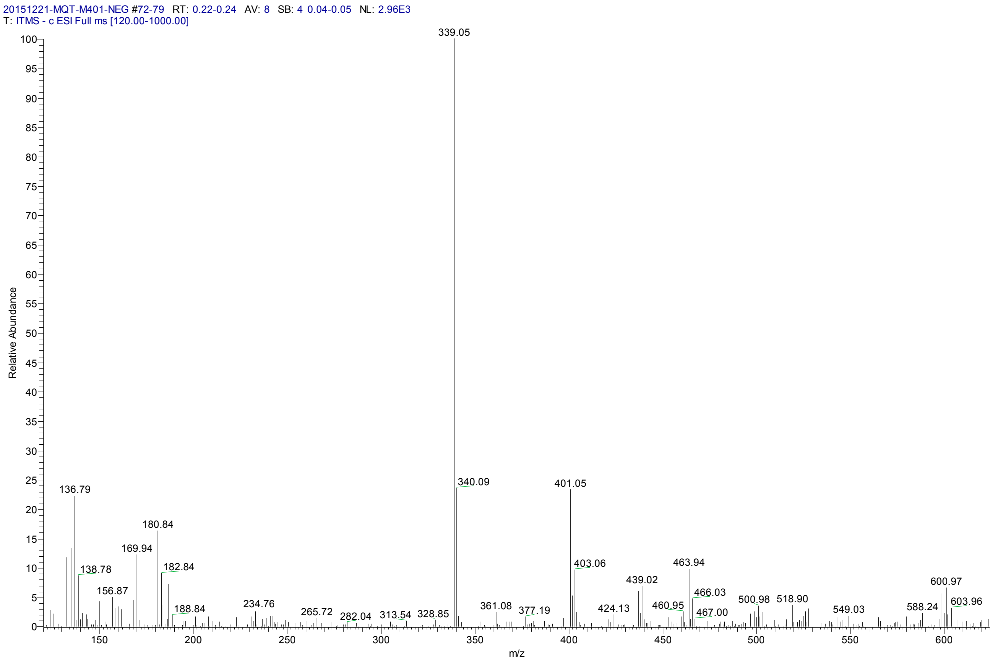

Figure S16. ESI-mass spectra of **HNA**-Cu^{2+} ensemble in the presence of Hcy.
